# Insulin resistance, metabolic syndrome and polycystic ovaries: an intriguing conundrum

**DOI:** 10.3389/fendo.2025.1669716

**Published:** 2025-10-01

**Authors:** Sara Prosperi, Francesco Chiarelli

**Affiliations:** Department of Pediatrics, University of Chieti-Pescara, Chieti, Italy

**Keywords:** polycystic ovary syndrome, PCOS, adolescent, type 2 diabetes mellitus, insulin resistance, obesity, metabolic syndrome, hyperandrogenism

## Abstract

Polycystic ovary syndrome (PCOS) is a multisystemic disorder and occurs as the most common endocrine condition in adolescent girls and young women. There is a strict interplay between PCOS and insulin resistance, obesity, and features of the metabolic syndrome; the link between these conditions is complex and often bidirectional: insulin resistance exacerbates hyperandrogenism and ovulatory dysfunction, and PCOS itself increases the risk of developing impaired glucose tolerance and type 2 diabetes mellitus (T2D). As the diagnosis of PCOS is mostly clinical, physicians need to be aware of the fact that, during adolescence, physiological insulin resistance of puberty and menstrual irregularity in the first years post menarche can complicate the diagnostic process, leading to both over- and under-diagnosis of PCOS. This review article explores the central role of insulin resistance as a unifying mechanism underlying both metabolic and reproductive dysfunction in young women, highlighting the overlapping clinical features, the difficulties in applying adult-based diagnostic criteria to adolescents, and the importance of identifying early red flags. Management requires a multidisciplinary approach that prioritizes lifestyle modification, psychological support, and, where needed, pharmacological interventions. Early recognition is critical to prevent long-term complications, including infertility, endometrial hyperplasia, and cardiovascular disease. Given the rising prevalence of insulin resistance, T2D and PCOS in youth, clinicians must become increasingly familiar with this metabolic and endocrine challenge in order to implement timely individualized care.

## 1 Introduction

### 1.1 Background

Polycystic ovary syndrome (PCOS) is the most prevalent endocrine condition among women of reproductive age (1). According to the 2003 Rotterdam consensus, PCOS can be described as a heterogeneous disorder of ovarian dysfunction characterized by the presence of at least two criteria of the following: ovulatory dysfunction (oligo- or anovulation, amenorrhea), hyperandrogenism (clinical or biochemical) and micropolycystic ovarian morphology on ultrasound (at least 20 follicles per ovary and/or ovarian volume >10 ml on either ovary) (2).

To date, however, there are no universally validated diagnostic criteria for PCOS tailored to adolescents. The Endocrine Society recommends a cautious approach, according to which a diagnosis of PCOS can be considered in the presence of persistent oligomenorrhea and evidence of hyperandrogenism (either clinical or biochemical) after the exclusion of other differential diagnoses (non-classic congenital adrenal hyperplasia, thyroid dysfunction, hyperprolactinemia…). The use of the Rotterdam criteria in adolescent populations remains debated due to the inclusion of polycystic ovarian morphology on ultrasound as in adolescents a multifollicular appearance of the ovaries can be considered a physiological finding, not necessarily reflecting pathology (3, 4).

In the past years, PCOS has been more frequently recognized as a syndrome rather than a disease, encompassing a spectrum of reproductive as well as metabolic abnormalities, and not relying on a single pathognomonic feature for its diagnosis. Common clinical manifestations include menstrual irregularities, hirsutism, acne, and, in many cases, obesity. Biochemically, insulin resistance and elevated luteinizing hormone (LH) levels are frequently observed, even in the absence of evident metabolic disease. Adolescents and women with PCOS face an increased lifetime risk of developing type 2 diabetes mellitus (T2D), cardiovascular complications, pregnancy complications (5–7).

The prevalence of PCOS is estimated to be 5%-18% in reproductive age women (8) and between 3%-11% in adolescents (9). Women with PCOS are at increased risk of progressing from insulin resistance to T2D, and several studies have reported a higher incidence of T2D in this population, particularly in association with elevated body mass index (BMI) (10). However, metabolic disturbances and glucose intolerance have also been documented in lean women with PCOS (11), suggesting that the syndrome itself, beyond excess weight, may contribute to altered hematological and metabolic profiles. These findings highlight the need for early detection and the importance of systematic screening for metabolic comorbidities in all patients with PCOS, regardless of their age and BMI. Given this complex metabolic and reproductive profile, it is increasingly clear that adolescents with PCOS cannot be simply considered as “younger adults”; developmental differences require age-specific approaches. Therefore, this review aims to provide a comprehensive synthesis of current evidence on the pathophysiology linking insulin resistance and PCOS in adolescents, as well as the associated comorbidities, by discussing the unique diagnostic challenges in this age group, the rationale for early metabolic screening, and the implications for timely intervention.

### 1.2 Systemic nature of PCOS and rationale for renaming

PCOS represents a complex, multisystem disorder that extends beyond the reproductive system, involving metabolic, cardiovascular, dermatological, and psychological aspects. Insulin resistance is a central pathophysiological feature, contributing not only to hyperandrogenism and ovulatory dysfunction but also to metabolic complications such as obesity, dyslipidemia, and impaired glucose tolerance (4, 12); these abnormalities may occur even in adolescents and lean individuals, emphasizing that metabolic imbalances are intrinsic to the syndrome rather than purely a consequence of excess weight. In addition to metabolic and reproductive concerns, PCOS is linked to considerable psychological morbidity, including anxiety, depression, and body image issues related to hirsutism, acne, and weight (13). These features are often underestimated, and the current terminology, emphasizing ovarian morphology rather than the syndrome’s multisystemic nature, is not helpful in increasing awareness regarding these aspects of the syndrome. In the past decade, the term “polycystic ovary syndrome” has been questioned for not reflecting the full range of clinical manifestations, potentially contributing to underdiagnosis and delayed recognition of complications (13, 14). A revised nomenclature could increase awareness among clinicians and patients, promote earlier diagnosis, and support a holistic approach to management that integrates reproductive, metabolic, hepatic, and psychological care.

Over the years, several alternative names have been proposed: “Estrogenic Ovulatory Dysfunction” or “Functional Female Hyperandrogenism” (15) were initially proposed to emphasize the hormonal and ovulatory abnormalities central to the syndrome, though they were considered too general and not capable of capturing the metabolic dimensions of the condition. Additionally, some authors have suggested a dual-naming approach to distinguish between PCOS phenotypes: individuals whose condition primarily manifests with reproductive consequences would retain the PCOS label, while those with predominant metabolic complications would be assigned a separate, metabolically oriented name (16). Other authors proposed the name “Ovarian Dysmetabolic Syndrome” to highlight the metabolic disturbances often accompanying PCOS (17). More recently, a large-scale international survey from Monash University involving over 7700 participants from all continents, revealed that 76% of health professionals and 86% of patients supported renaming PCOS, arguing that the current name misrepresents the complexity of the condition and contributes to stigma. There is now an ongoing survey regarding new names for PCOS. Among the options being considered are names that emphasize both reproductive and metabolic aspects; the option of retaining the acronym PCOS redefining the meaning of each letter is also explored. The options currently under consideration are *Polyendocrine Cardiometabolic Ovulatory Syndrome (PCOS)*, *Metabolic Reproductive Syndrome (MERS)*, *Reproductive Metabolic Syndrome (RMS)*, *Metabolic Reproductive Endocrine Syndrome (MRES)*, *Chronic Polyendocrine Syndrome (CPS)*, *Polyendocrine Complex Ovulatory Syndrome (PCOS)*, *Polyendocrine Chronic Ovulatory Syndrome (PCOS)*, *Endocrine Metabolic Reproductive Syndrome (EMRS)*, and *Endocrine Reproductive Metabolic Syndrome (ERMS)* (18). A revised nomenclature could improve awareness among healthcare providers and patients, promote earlier diagnosis, and encourage a holistic approach to management.

## 2 The central role of insulin resistance

Insulin resistance represents a common pathophysiological substrate linking PCOS, metabolic syndrome, and T2D. While traditionally viewed as distinct entities, they share overlapping mechanisms of altered insulin signaling, adiposity-related inflammation, and hormonal dysregulation.

**Figure 1:**
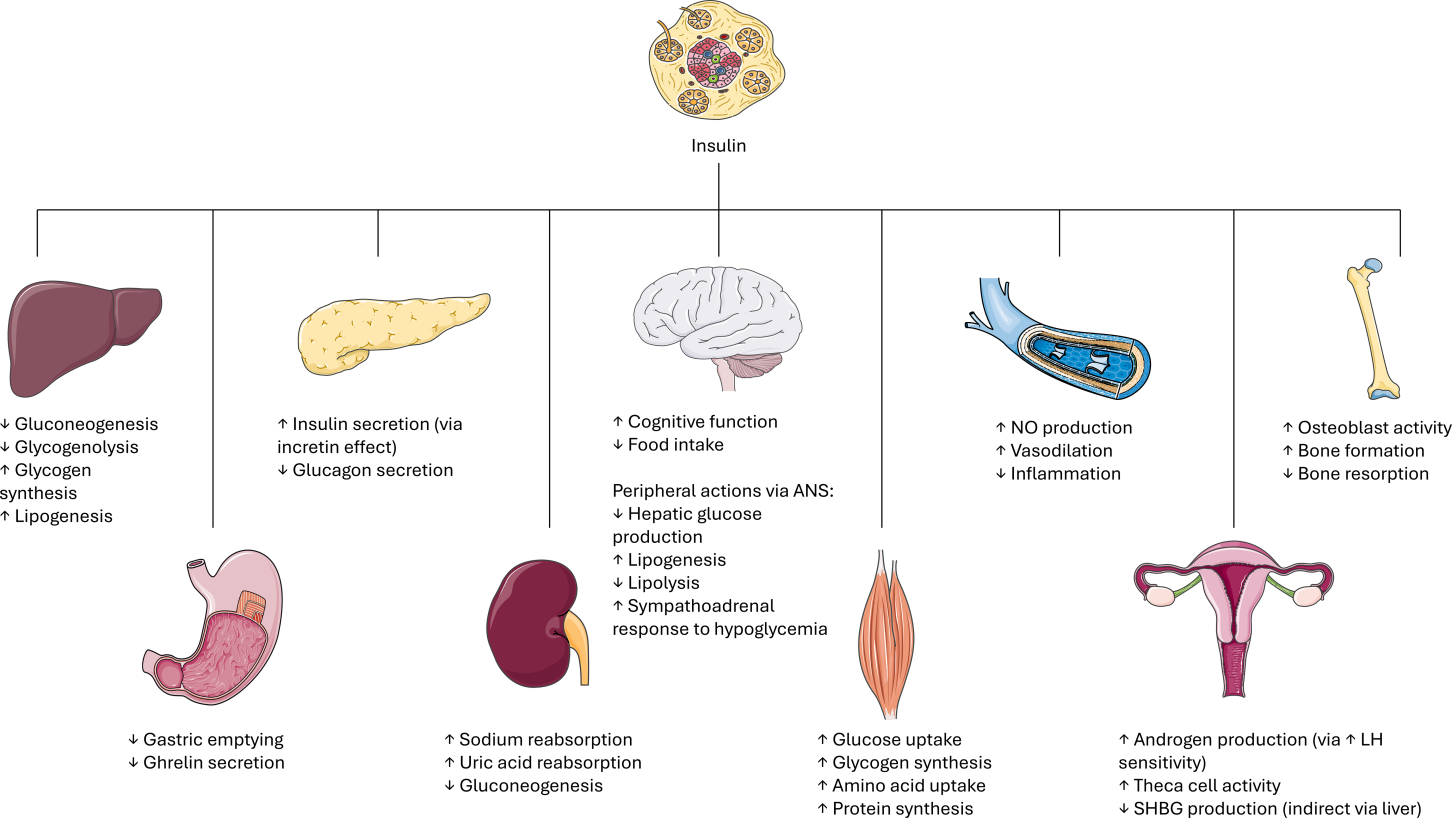
Schematic overview of insulin’s actions across key organs and tissues. Insulin exerts pleiotropic effects on multiple target tissues to coordinate energy metabolism, growth, and reproduction. ANS: autonomic nervous system. NO: nitric oxide. LH: luteinizing hormone. SHBG: sex hormone binding globulin. Image adapted from Servier Medical Art (https://smart.servier.com/), licensed under CC BY 4.0 (https://creativecommons.org/licenses/by/4.0/).

Insulin is the principal regulator of glucose, inducing its uptake in all tissues, particularly adipose tissue, muscle, heart, liver, and maintaining its homeostasis. Insulin has also a role in decreasing lipolysis and modulating the levels of blood free fatty acids. Another important role played by insulin is as a co-gonadotropin: it magnifies the action of LH on theca cells, participating in androgen secretion from the ovaries, and has a role in the production of the sex hormone binding globulin (SHBG) and in dehydroepiandrosterone (DHEA) release from the adrenals (19, 20). **Figure 1** provides a schematic overview of insulin’s pleiotropic actions across key organs and tissues, highlighting how insulin coordinates not only glucose uptake and lipid metabolism in peripheral tissues, but also influences reproductive function through modulation of LH signaling in the ovaries and SHBG production. Additionally, the figure illustrates insulin’s interactions with the autonomic nervous system (ANS) and its effect on nitric oxide (NO) pathways, emphasizing the hormone’s integrative role in linking energy metabolism, growth, and reproductive regulation (**Figure 1**).

In adolescents, particularly females, this intersection is clinically relevant, as puberty marks a physiological reduction in insulin sensitivity. During puberty, insulin sensitivity naturally decreases by approximately 30%, reaching its nadir in mid-puberty, and recovering post-menarche. This physiological insulin resistance is driven by the pubertal rise in growth hormone and sex steroids, which increase lipolysis and reduce insulin action at the skeletal muscle level. Normally, pancreatic β-cells compensate by increasing insulin secretion (21). However, in predisposed individuals, such as those with PCOS, this compensation is often insufficient or maladaptive.

In adolescents with PCOS, insulin resistance is often independent of weight status, suggesting intrinsic defects in insulin receptor signaling. During past decades, several mechanisms have been proposed and include post-receptor insulin signaling abnormalities, particularly in the PI3K/Akt pathway (influencing the expression of glucose transporter-4, GLUT-4) (22), increased serine phosphorylation of insulin receptor substrate-1 (IRS-1) (23), adipocyte dysfunction with increased release of pro-inflammatory cytokines (TNF-α, IL-6), mitochondrial dysfunction and oxidative stress (24). All these defects reduce glucose uptake in peripheral tissues and promote hepatic gluconeogenesis, contributing to hyperglycemia and compensatory hyperinsulinemia.

Hyperinsulinemia is not merely a compensatory response, but it is one of the fundamental endocrine features of PCOS: in ovarian theca cells, insulin acts together with LH to enhance androgen biosynthesis; insulin also reduces hepatic production of SHBG, thus increasing circulating free androgens. This results in the key clinical features of PCOS: hirsutism, acne, and ovulatory dysfunction. In many adolescent girls with PCOS insulin resistance, rather than being a consequence of androgen excess, may precede and lead to the endocrine abnormalities of PCOS itself.

The prevalence of insulin resistance in PCOS women and adolescents is quite high, ranging from 35% to 80%, and women with PCOS and obesity are more frequently insulin resistant than nonobese controls (3, 25). Insulin resistance and the consequent hyperinsulinemia are early features in the pathophysiology of PCOS, and this early onset was clearly demonstrated in literature. In a cohort of obese adolescents diagnosed with PCOS, with an average age of 12 ± 0.7 years, girls exhibited a marked impairment in insulin sensitivity, with an average 50% reduction in peripheral insulin responsiveness compared to a control group of obese girls without PCOS. Hepatic insulin resistance was also noted. This metabolic dysfunction was accompanied by compensatory hyperinsulinemia, reflecting the body’s attempt to maintain glucose homeostasis despite reduced insulin action (26).

T2D that begins during youth appears with greater severity compared to adult-onset diabetes: adolescents with T2D experience a more rapid progression of the disease, with higher rates of complications, and shorter life expectancy than adults living with diabetes (27). Moreover, standard treatments for T2D including lifestyle modifications, metformin, insulin, and thiazolidinediones tend to show higher failure rates reported in this population (28). While adult women PCOS are known to have an increased risk of developing T2D, there remains a significant gap in evidence-based data regarding the exact incidence of T2D among adolescent girls, so further research is needed to clarify this risk and guide early intervention efforts.

## 3 The association between PCOS and metabolic syndrome

The diagnostic criteria for metabolic syndrome vary across international organizations and have evolved over time, leading to differences in prevalence estimations and clinical interpretation. While most definitions include common components such as central obesity, dyslipidemia, elevated blood pressure, and impaired glucose regulation, they differ in terms of specific thresholds, and whether certain elements (such as insulin resistance or central obesity) are mandatory. For example, the WHO criteria require evidence of insulin resistance, defined as type 2 diabetes, impaired glucose tolerance (IGT), impaired fasting glucose (IFG), or reduced insulin sensitivity, plus any two additional components such as hypertension, dyslipidemia, central obesity, or microalbuminuria (29). In contrast, the ATP III criteria define metabolic syndrome as the presence of any three or more of five risk factors, including elevated waist circumference, high blood pressure, elevated triglycerides, low HDL cholesterol, and elevated fasting glucose, without making insulin resistance a prerequisite (30). The IDF criteria further emphasize central obesity as an essential component, with ethnicity-specific waist circumference cut-offs, while also requiring at least two additional risk factors for diagnosis (31). These differences in mandatory components, thresholds for glucose, blood pressure, and lipid levels can substantially influence the estimated prevalence and identification of at-risk individuals. In pediatric populations, the challenge is even greater due to age-, sex-, and pubertal stage-related variations in metabolic parameters. Consequently, several pediatric definitions have been proposed, yet no single consensus has been universally adopted. This variability can complicate both clinical diagnosis and cross-study comparison. Nevertheless, from the age of 16 onward, adolescents are frequently assessed using adult criteria (**Table 1**).

**Table 1:** Comparison of diagnostic criteria for metabolic syndrome.

Component	WHO (1999)	ATP III (2001)	IDF (2005)
	Insulin resistance is required plus any 2 of the following	Any 3 or more of the following 5 criteria	Central obesity is required + any 2 of the following
Insulin Resistance	Mandatory: Type 2 diabetes, IGT, IFG, or low insulin sensitivity	*Not required*	*Not required*
Central Obesity	Waist-to-hip ratio > 0.85 (women) or BMI > 30 kg/m²	Waist circumference > 88 cm (women)	Mandatory: Waist circumference ≥ 80 cm (women, Europid); ethnicity-specific
Dyslipidemia	Triglycerides ≥ 150 mg/dL or pharmacological treatment	Triglycerides ≥ 150 mg/dL or pharmacological treatment	Triglycerides ≥ 150 mg/dL or pharmacological treatment
	HDL cholesterol < 39 mg/dL or pharmacological treatment	HDL cholesterol < 50 mg/dL or pharmacological treatment	HDL cholesterol < 50 mg/dL or pharmacological treatment
Blood Pressure	≥ 140/90 mmHg or receiving treatment for hypertension	≥ 130/85 mmHg or receiving treatment for hypertension	≥ 130/85 mmHg or treatment for hypertension
Glucose Abnormalities	IGT, IFG, or diabetes (part of the insulin resistance definition)	Fasting plasma glucose ≥ 110 mg/dL (revised to ≥ 100 mg/dL in 2005)	≥ 100 mg/dL or previously diagnosed type 2 diabetes
Microalbuminuria	Urinary albumin excretion ≥ 20 µg/min or albumin-to-creatinine ratio ≥ 30 mg/g	*Not included*	*Not included*

IGT, impaired glucose tolerance; IFG, impaired fasting glucose; HDL, high density lipoprotein.

The differences are particularly relevant when evaluating the prevalence of metabolic syndrome in specific subgroups. In adolescent girls with PCOS and subsequently in women with PCOS, particularly in those with obesity, the prevalence of metabolic syndrome is elevated. In a comparative analysis, overweight and metabolic syndrome were observed in 52% and 33.3% of PCOS adolescents, significantly higher than in controls; among all subgroups, obese adolescents with PCOS showed the highest prevalence of insulin resistance (61.5%), hypercholesterolemia (46.2%), central obesity (53.8%), and metabolic syndrome (69.2%) (32).

Another study assessed the prevalence of metabolic syndrome in 282 Italian women with PCOS aged 18–40 years (33), using both the ATP-III and WHO criteria. The study found a significantly lower prevalence of metabolic syndrome in this population (8.2% by ATP-III and 16% by WHO) compared to reports from the USA. The prevalence was higher in women with classic PCOS than in those with ovulatory PCOS, and body weight was a key modifying factor, suggesting that differences in lifestyle, diet, and genetics may influence the expression of metabolic abnormalities in PCOS.

A study evaluating the metabolic profile of adolescents with PCOS diagnosed according to the NIH 1990 criteria compared 30 PCOS adolescents with 71 healthy controls. The results showed that adolescents with PCOS had a significantly higher prevalence of overweight (52%) and metabolic syndrome (33.3%) compared to controls (22.4% and 11.26%, respectively). Among all subgroups, obese adolescents with PCOS exhibited the most unfavorable metabolic profile, with markedly increased rates of insulin resistance (61.5%), hypercholesterolemia (46.2%), central obesity (53.8%), and metabolic syndrome (69.2%) (32).

Taken together, these findings highlight the strong association between PCOS and metabolic syndrome, particularly in the presence of obesity. Obese adolescents and women with PCOS consistently demonstrate a higher burden of cardiometabolic risk factors, including insulin resistance, dyslipidemia, central adiposity, and elevated blood pressure.

However, it is important to recognize that metabolic dysfunction is not limited to obese individuals. Several studies have shown that even nonobese adolescents and women with PCOS may present with insulin resistance, altered lipid profiles, and other early markers of metabolic impairment. This suggests that PCOS itself, independently of body weight, may confer an underlying risk for metabolic abnormalities.

However, although obesity is a well-known contributor to insulin resistance and T2D, increasing evidence suggests that metabolic dysfunction is not limited to obese individuals: all women with PCOS may develop metabolic disturbances regardless of their body weight. Several studies have shown that nonobese adolescents and women with PCOS can present with insulin resistance, altered lipid profiles, impaired glucose metabolism, compensatory hyperinsulinemia, and other early markers of metabolic impairment, and this indicates that PCOS itself may be the factor which gives an intrinsic risk for metabolic abnormalities.

A large Australian population-based study (2000–2015) showed that women with PCOS have a significantly higher risk of developing type 2 diabetes, independently of BMI, with an incidence rate of diabetes over four times higher in women with PCOS compared to controls. In this study, while obesity increased the absolute risk, the relative risk linked to PCOS was even more pronounced in lean women, highlighting the role of PCOS as a strong independent predictor of diabetes (34).

A systematic review and meta-analysis comparing nonobese women with PCOS to healthy nonobese controls revealed that PCOS is associated with significantly higher chances of metabolic disturbances such as insulin resistance, impaired glucose tolerance, hyperinsulinemia, T2D, hypertriglyceridemia, low HDL. Subgroup analyses showed these risks were more evident in white populations rather than in Asian cohorts (35).

Studies conducted in East Asian populations also seem to confirm that nonobese women with PCOS have a significantly increased hazard ratio for developing T2D compared to non-PCOS controls; unlike Western populations, the majority of PCOS patients in Asian cohorts had a normal BMI, making the findings particularly relevant to East Asian populations (36).

These conclusions imply that PCOS itself can be an independent risk factor for metabolic disorders, with insulin resistance being driven not only by adiposity, but also by intrinsic abnormalities in insulin signaling, adipokine secretion, and androgen excess. Several cohort studies and meta-analyses have identified higher incidence rates of impaired glucose tolerance and T2D in lean women with PCOS too, although with slightly lower risk compared to their obese counterparts.

A significant limitation shared by most of the available studies is that they have been mainly conducted in adult women, with a shortage of data specifically addressing adolescent populations, and this represents an important gap in current knowledge considering that many features of PCOS, such as hyperandrogenism and menstrual irregularities, often emerge during adolescence. The diagnostic complexity in this age group, combined with the evolving endocrine and metabolic physiology of puberty, makes it challenging to extrapolate adult findings directly to younger cohorts. Despite this, the consistent evidence from adult studies demonstrates a clear association between PCOS and an increased risk of metabolic disturbances even in nonobese individuals, and those findings are sufficient to underscore the critical need to adopt a proactive approach to metabolic screening in all individuals diagnosed with PCOS, beginning as early as possible and across the entire BMI spectrum.

## 4 Gut microbiome and metabolic dysregulation

The terms microbiota and microbiome are related and often used interchangeably but refer to distinct concepts. The microbiota describes the community of microorganisms living in a specific environment, such as the human gut, including bacteria, viruses, fungi, protozoa, and archaea, whereas the microbiome encompasses not only these microorganisms but also their collective genomes, better reflecting their functional capacities and interactions with the host. The human gut microbiome is composed of trillions of microorganisms, dominated by bacterial phyla such as *Bacteroides* and *Firmicutes*, followed by *Proteobacteria*, *Fusobacteria*, and *Actinobacteria* (37). This complex ecosystem contributes to numerous processes, including nutrient metabolism, immune modulation, and maintenance of the intestinal barrier. Emerging evidence indicates that the gut microbiome also plays a central role in the metabolic disturbances observed in PCOS.

Individuals with insulin resistance show an imbalance of their gut microbiota with different representation of *Ruminococcaceae* and *Lachnospiraceae*, and that facilitates an increase in intestinal permeability, consequent chronic low-grade inflammation linked to the continue activation of the immune system and, lastly, an augmented production of inflammatory cytokines that seem to interfere with the insulin receptor (38, 39). In addition, some studies reported that an increase in *Bacteroides* species can lead to alterations in ghrelin and peptide YY secretion, causing a progression towards hyperinsulinemia and insulin resistance (40, 41). Dysbiosis can also ease alterations in bile acid metabolism, which in turn affects insulin sensitivity and lipid metabolism, and lead to a reduced short-chain fatty acids (SCFAs) production, exacerbating metabolic imbalances (42, 43). Finally, there is evidence regarding the influence of microbiome on sex hormones. In particular, the microbiome of healthy women is characterized by greater variance and lower presence of *Bacteroides* compared to that of men. Elevated levels of *Bacteroides*, *Escherichia*, *Shigella*, and *Streptococcus* appear to correlate with increased testosterone concentrations, suggesting a potential role of the gut microbiota in androgen regulation (40, 44).

To further support these results, studies in women with PCOS have shown that probiotic supplementation has demonstrated positive effects on various metabolic and hormonal parameters. A recent systematic review and meta-analysis of randomized controlled trials reported significant reductions in weight, BMI, fasting plasma glucose, insulin levels, lipids and total testosterone levels in women with PCOS who underwent probiotic supplementation (45). Similarly, a systematic review and meta-analysis found that supplementation with probiotics, prebiotics, or synbiotics led to better metabolic profile in PCOS women (46). Synbiotics, which combine probiotics and prebiotics, were found to have more pronounced effects compared to probiotics or prebiotics alone.

However, it is important to note that some of these studies present limitations. Notably, a randomized trial (47), which initially reported improvements in weight, glycemia, and lipid profiles following probiotic supplementation for 12 weeks after discontinuation of other PCOS medications at least three months prior, was later retracted due to concerns regarding the integrity of the data and trial design, rendering the reported conclusions unreliable (48).

More generally, studies in this field vary in terms of methodology, probiotic strains, doses, duration of supplementation, and sometimes include relatively small sample sizes. Nevertheless, preliminary evidence is promising and opens interesting therapeutic perspectives, including probiotic or synbiotic supplementation, fecal microbiota transplantation, and modulation of intestinal immune factors such as IL-22 (49). These approaches may offer novel strategies to improve metabolic, inflammatory, and hormonal parameters in PCOS, but additional longitudinal studies and larger randomized controlled trials are much needed in the future to provide more data and guide clinical application.

## 5 Metabolic risk assessment

**Figure 2:**
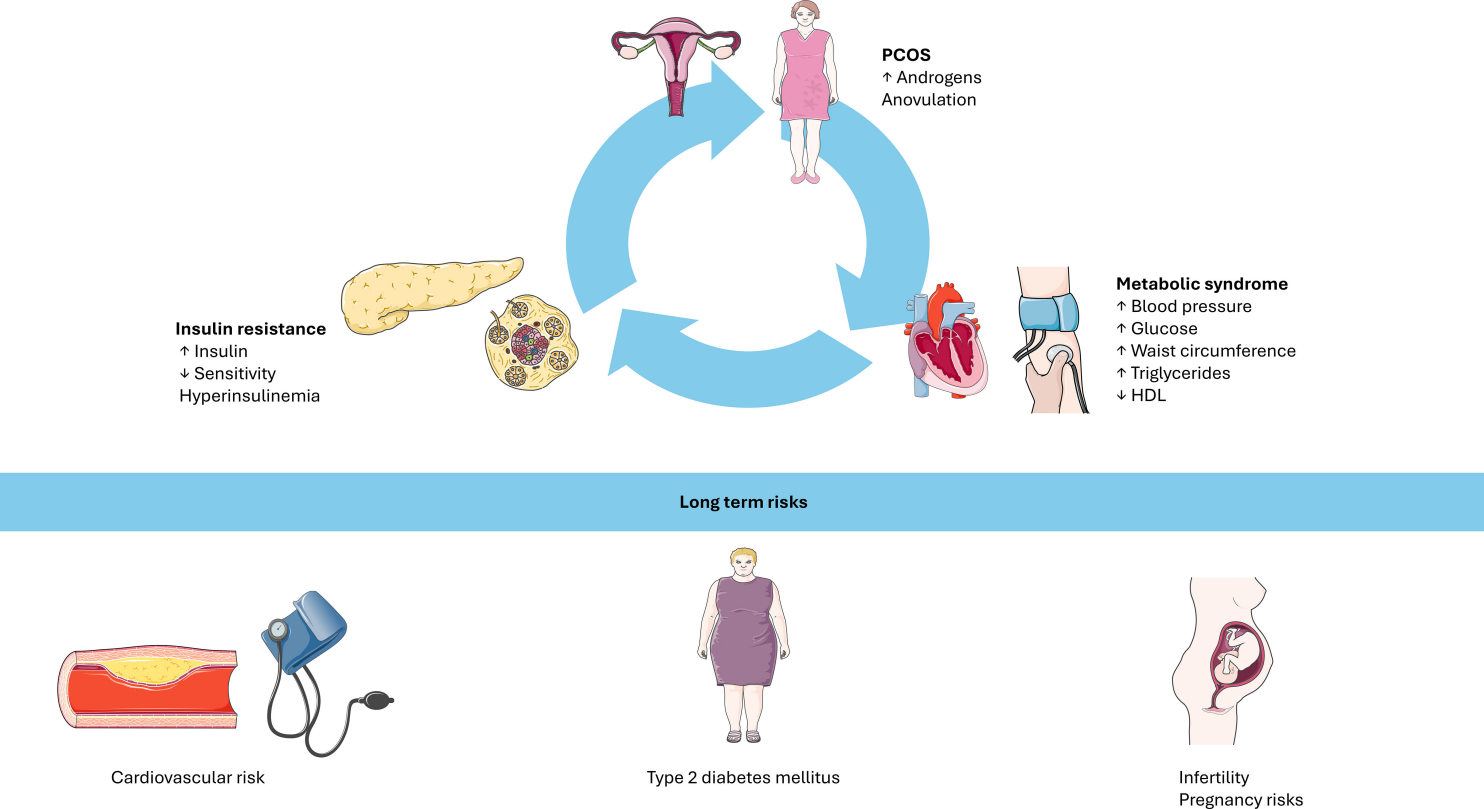
The interplay between PCOS, insulin resistance, and metabolic syndrome forms a self-perpetuating vicious cycle. Hyperinsulinemia exacerbates ovarian hyperandrogenism, while insulin resistance contributes to metabolic dysfunction. Even lean individuals with PCOS may display significant metabolic risk. Long-term consequences include cardiovascular disease, type 2 diabetes, infertility, and pregnancy complications. PCOS: polycystic ovary syndrome. HDL: high density lipoprotein. Image adapted from Servier Medical Art (https://smart.servier.com/), licensed under CC BY 4.0 https://creativecommons.org/licenses/by/4.0/).

Given the pathophysiological continuum linking PCOS, insulin resistance, T2D, obesity and metabolic syndrome (**Figure 2**), early identification and intervention are required. The relationship between PCOS, insulin resistance, and metabolic syndrome forms a self-perpetuating vicious cycle. Hyperinsulinemia, a hallmark of insulin resistance, amplifies ovarian androgen production, contributing to hyperandrogenism and to the clinical manifestations of PCOS. Such metabolic disturbance, in turn, further impairs insulin sensitivity, promoting dyslipidemia, altered glucose metabolism, and other features of metabolic syndrome. Importantly, the figure emphasizes that these processes are not confined to overweight or obese individuals; lean adolescents with PCOS can also exhibit significant metabolic risk. This normal weight but metabolically unhealthy phenotype underscores the need for comprehensive screening in all adolescents considered at risk.

One of the primary challenges in assessing metabolic risk in adolescents with PCOS is the lack of standardized, pediatric-specific guidelines. Current screening strategies are extrapolated from studies conducted in adult women with PCOS and are often applied to adolescent populations despite physiological differences. While several peer-reviewed studies have compared the diagnostic performance of various tests in identifying impaired glucose tolerance, impaired fasting glucose, T2D, and insulin resistance in adults, pediatric data remain limited. As a result, the sensitivity and specificity of these tests in the adolescent population are not well established, and their predictive value for metabolic disturbances in this age group remains uncertain. Nevertheless, in the absence of pediatric-specific recommendations, current clinical practice often relies on adult guidelines for screening decisions in adolescents with PCOS.

### 5.1 Direct markers of insulin resistance

There are many methods and biomarkers to check for insulin resistance. The gold standard method would be the hyperinsulinemic euglycemic clamp, used in studies to assess β-cell sensitivity (50). This method, however, requires extensive procedures, time and expertise, and has no relevance for daily clinical practice. Since the clamp is not applicable in large investigations and in daily practice, the need for surrogate markers arises.

### 5.2 Surrogate markers of insulin resistance: anthropometric markers

Among anthropometric markers used to estimate insulin resistance, BMI and waist circumference are the most commonly used. However, they are both limited by the fact that insulin resistance in PCOS is often independent of adiposity, especially in lean women. Waist-to-hip ratio is judged less accurate and has largely been abandoned in clinical practice. Waist-to-height ratio appears more consistent than BMI and waist circumference in predicting metabolic and cardiovascular risks, though data remain limited (51). Notably, wrist circumference, a bone-based marker, has emerged as the most accurate anthropometric measure of insulin resistance in both lean and obese PCOS women. Its utility lies in its strong correlation with insulin resistance independent of fat mass: osteocalcin, a hormone secreted by osteoblasts, plays an endocrine role in glucose homeostasis by enhancing insulin sensitivity. In states of insulin resistance, compensatory hyperinsulinemia has been linked to increased bone mass, which can be indirectly assessed through wrist circumference (52, 53). Despite these premises, wrist circumference as a parameter of insulin resistance is underrepresented in the literature and warrants further validation.

### 5.3 Surrogate markers of insulin resistance: biological markers

As for biological markers, the first question regards whether glycated hemoglobin (HbA1c) could be a suitable tool to screen for insulin resistance. Recommended by the American Diabetes Association to screen for diabetes, HbA1c is not affected by day-to-day fluctuations of plasma glucose levels, as it reflects the plasma glucose mean status during the 2–3 months prior the measurement. But when used as a screening method to predict insulin resistance and other complications in PCOS patients, some disagreements emerge: in some studies HbA1c failed to identify insulin resistance, while HOMA or fasting insulin levels did (51, 54).

Another fast, simple method consists of measuring fasting serum insulin; nevertheless, it should be noted that its levels can be normal in a big percentage of PCOS women with impaired glucose tolerance diagnosed via the oral glucose tolerance test (OGTT), thus failing to identify dysglycemia in an early stage (55).

The 2-hour 75 g OGTT was treated as the reference standard for identifying impaired glucose tolerance, given its ability to find out postprandial hyperglycemia not captured by fasting plasma glucose or HbA1c measurements. Several studies directly compared OGTT results with HbA1c values in adolescents with PCOS, stratifying findings by BMI percentile and metabolic phenotype. Among these works, some reported that adolescents with a BMI above the 95th percentile demonstrated a significantly higher prevalence of glucose abnormalities, supporting routine OGTT screening in this group (54, 56, 57). Conversely, one study found that OGTT granted limited diagnostic benefit in adolescents with PCOS and BMI <85th percentile, raising concerns about overtesting and cost-efficiency in this subgroup of lean PCOS girls (57).

Various indices have been developed to assess insulin resistance in clinical and research settings. They are typically derived from fasting blood samples or from values obtained during the OGTT, and they aim to provide practical, simple alternatives. The Homeostasis Model Assessment (HOMA), first introduced in 1985, was designed to evaluate insulin resistance and β-cell function from basal glucose and insulin levels (58). Using mathematical modeling, the authors derived reference values of glucose and insulin for variable degrees of insulin resistance and β-cell function. The most commonly used formula to estimate insulin resistance is: HOMA-IR=(fasting insulin [µU/mL] × fasting glucose [mmol/L])/22.5. HOMA-IR has shown strong correlation with the euglycemic clamp and has been broadly used in studies of women with PCOS across various populations (59–61). To enhance its performance, a log transformation of HOMA-IR, known as log(HOMA-IR) or Ln(HOMA-IR), has been proposed, but its use in PCOS populations remains limited.

Another work investigates a novel insulin sensitivity index, the HOMA-M120, derived from OGTT values and calculated as: HOMA-M120=(Insulin_120min [mU/L] × Glucose_120min [mmol/L])/22.5. Its validity has been assessed as a surrogate for insulin sensitivity in a population of women with PCOS, comparing it with the euglycemic hyperinsulinemic clamp and with other commonly used indices. The results showed that the HOMA-M120 index had a stronger correlation with the clamp-derived insulin sensitivity index thus serving as a simple, cost-efficient, and accurate surrogate marker for insulin resistance in clinical and research settings focused on PCOS. This index also showed to have a great reliability in lean women with PCOS (62, 63).

A further index is the Fasting Insulin Resistance Index (FIRI), introduced in 1989, and calculated as: FIRI=(fasting glucose × fasting insulin)/25 (64). FIRI index has been infrequently used in PCOS studies.

The Quantitative Insulin Sensitivity Check Index (QUICKI) offers another simplified surrogate of insulin sensitivity and is calculated as: QUICKI=1/[log(fasting insulin [µU/mL]) + log(fasting glucose [mg/dL])]. QUICKI has been validated as a valid alternative to the euglycemic clamp, particularly in obese and diabetic populations, but its reliability is reduced in nonobese individuals (65).

Finally, indices derived from OGTT values may provide more accurate estimations of insulin sensitivity, especially in populations where fasting-based markers underperform. The Matsuda Index utilizes multiple glucose and insulin values during OGTT to estimate whole-body insulin sensitivity (66). In women with PCOS, it has shown strong correlation with both HOMA-IR and QUICKI, and has emerged as a reliable alternative for detecting IR (67).

Similarly, the Stumvoll Index, the Avignon index, and the Gutt index calculate insulin sensitivity using OGTT-derived values, but there are few studies regarding those indices in PCOS women.

### 5.4 Emerging markers

Recent studies have stressed the importance of identifying early metabolic alterations in adolescents with PCOS to better predict long-term health outcomes. Among various biomarkers, lipid profile parameters are gaining attention as indicators of metabolic risk (even in lean PCOS women).

An interesting recent study offers preliminary evidence on the application of markers such as the triglyceride to high density lipoprotein (HDL) cholesterol (TG/HDL-C) ratio to provide valuable insight into early metabolic changes in PCOS population. Notably, abnormalities in HDL-C levels and TG/HDL-C ratios identified during adolescence tend to persist into adulthood, even in the absence of significant weight gain, and these remarks suggest that dyslipidemia may be an intrinsic characteristic of PCOS, independent of obesity or insulin resistance (68).

The TyG index, calculated as (ln [fasting triglycerides × fasting glucose/2]), has emerged as a simple and practical surrogate marker of insulin resistance. Evidence shows that higher TyG values are associated with adverse metabolic profiles, including increased risk of dyslipidemia and cardiovascular events (69). In women with PCOS, studies consistently report elevated TyG values compared with controls, suggesting that this index may be useful for metabolic risk stratification rather than as a standalone diagnostic tool for insulin resistance. Fifteen studies including over 7000 participants consistently found that the TyG index is higher in women with PCOS compared to controls. Subgroup analyses confirmed this association in different populations (from China, Turkey, Iran, Sudan, Poland, Indonesia and Korea) and study designs (70). The TyG index showed excellent accuracy for identifying PCOS patients with metabolic syndrome. Its calculation requires only routine fasting glucose and triglyceride measurements, making it feasible in daily clinical practice, although specific cut-offs tailored for PCOS populations remain to be established.

Another work has reported elevated levels of inflammatory markers, including cystatin C, high-sensitivity C-reactive protein (hs-CRP), and the neutrophil-to-lymphocyte ratio (NLR) in young women with PCOS, regardless of their BMI. Cystatin C is a secreted inhibitor of cysteine proteases. Dysregulated cysteine protease activity has been implicated in several pathological processes, including inflammation and tumor dissemination, and has recently emerged as a potential cardiometabolic risk marker in women with PCOS. H-CRP, predominantly produced in the liver, represents the most sensitive acute-phase reactant; its hepatic synthesis is driven by proinflammatory cytokines such as interleukin-1, interleukin-6, and tumor necrosis factor. Data from this study suggest that low-grade chronic inflammation may play a role in the pathophysiology of PCOS and contribute to its associated metabolic risk: consequently, inflammatory markers such as hs-CRP and cystatin C have been proposed as potential tools for early identification of individuals with PCOS at higher risk of cardiometabolic complications (71).

The relation between serum cystatin C levels and PCOS has been explored in other studies (72, 73). Previous research showed that serum cystatin C levels are elevated in adolescents with PCOS, independent of blood pressure, lipid profile, or demographic variables, and so suggesting a role beyond that of a marker of renal function. Notably, the Authors also reported subclinical coronary atherosclerosis in adolescents with PCOS even in the absence of manifest cardiovascular risk factors. Based on these results, cystatin C may represent an early indicator of adverse cardiometabolic outcomes in this population, in conjunction with elevated BMI, HOMA-IR, triglycerides, and cholesterol (73). This result supports the hypothesis that cystatin C may be an indirect marker of chronic, low-grade inflammation and be associated with increased risk for insulin resistance and cardiovascular disease in adolescents with PCOS.

Despite promising associations, the applicability of these biomarkers remains limited and issues such as low specificity, lack of standardized cutoff values, and relatively high cost or limited availability of certain assays limit their routine use.

In terms of laboratory practicality, several of these biomarkers are straightforward to measure and widely available. The TG/HDL-C ratio and TyG index are both easily calculated from standard fasting lipid panels and glucose, making them inexpensive, rapid, and reproducible in most clinical laboratories without requiring specialized assays (74, 75). In contrast, hs-CRP is routinely available but requires a high-sensitivity assay; while common in many hospital labs, it is primarily intended for cardiovascular risk assessment. Cystatin C, although measurable with standardized immunoassays, is less commonly used in routine practice and may not be available in all centers; moreover, its clinical interpretation in the context of PCOS remains investigational. Overall, from a laboratory feasibility standpoint, TG/HDL-C and TyG stand out as the most practical non-insulin–based biomarkers, supporting their potential use. Overall, more studies are needed to determine whether these indicators can consistently distinguish high-risk PCOS phenotypes and guide personalized interventions, but, until then, their use should be considered exploratory and complementary to established assessments.

In addition, recent research increasingly supports the potential of microbiome-derived biomarkers in PCOS, reflecting both the composition and functional activity of the gut microbial ecosystem. Specific microbial taxa, as well as their metabolic products, have been linked to insulin resistance, low-grade inflammation, and hyperandrogenism. In clinical practice, such information might help identify patients who could benefit from targeted interventions, including specific probiotic or prebiotic supplementation, dietary modifications, or experimental approaches like fecal microbiota transplantation. However, microbiome-based biomarkers are not yet easy to implement in the laboratory; in addition, while these applications seem promising, widespread clinical implementation still requires measurement protocols, validation in larger cohorts and standardized thresholds (76).

## 6 Screening

The strong association between PCOS and various metabolic and endocrine disturbances including insulin resistance, metabolic syndrome, and increased cardiovascular risk, emphasizes the importance of comprehensive metabolic screening in this population. Early identification of these abnormalities is vital to implement timely non-pharmacological and pharmacological interventions, potentially reducing the risk of long-term complications such as T2D and cardiovascular disease. Evidence indicates that metabolic dysfunction is not confined to overweight or obese individuals with PCOS. In fact, lean women with PCOS may also exhibit insulin resistance and other metabolic alterations, supporting the need for universal metabolic risk assessment regardless of BMI.

Given the high prevalence of impaired glucose tolerance and T2D in women with PCOS, it has been proposed that the diagnostic threshold for initiating OGTT might need to be lower in this subgroup than in the general population (77). Furthermore, current guidelines recommend that all women with PCOS undergo periodic OGTT, even in the absence of classic risk factors. In cases where dysglycemia is detected, further evaluation of the lipid profile including triglycerides, total cholesterol, low density lipoprotein (LDL) cholesterol (LDL-C), HDL-C, and lipoprotein subfractions should be conducted, independently of BMI. As literature in adults suggests that non-alcoholic fatty liver disease (NAFLD) can be a predictor of future T2D and given the fact that NAFLD and T2D share similar pathophysiological mechanisms (78, 79), this broader metabolic assessment in PCOS women is essential for identifying high-risk phenotypes and guiding preventive strategies.

Screening recommendations for glucose abnormalities in women with PCOS vary among international guidelines, particularly in terms of preferred tests and screening intervals (**Table 2**). Current American Dietetic Association (ADA) and American College of Obstetricians and Gynecologists (ACOG) guidelines state that, as women with PCOS and obesity are classified as high risk for T2D, they should undergo annual screening for the development of diabetes if HbA1c is ≥5.7%, or every 3 to 5 years if HbA1c is within the normal range (80, 81). In contrast, the Endocrine Society clinical practice guidelines explicitly recommend using OGTT over HbA1c or fasting glucose for screening glucose abnormalities in women with PCOS, particularly those with a BMI ≥25 or other risk factors, due to the low sensitivity of HbA1c in detecting impaired glucose tolerance especially in adolescents (82). The International PCOS Guidelines (2018) also recommend using an OGTT in all adult women with PCOS who are overweight or obese, and suggest considering it in lean women with additional risk factors (14). However, these guidelines do not provide specific recommendations for adolescents, despite growing evidence of early-onset insulin resistance and cardiometabolic abnormalities.

**Table 2:** Comparison of glucose metabolism screening recommendations for women PCOS according to major professional societies.

Organization	Target population	Preferred screening method	Screening frequency
ACOG (American College of Obstetricians and Gynecologists)	Women with PCOS and obesity	HbA1c or OGTT	Annually if HbA1c ≥ 5.7%; every 3–5 years if normal
ADA (American Diabetes Association)	Women with PCOS (especially if obese)	HbA1c or OGTT	Every 1–3 years depending on HbA1c and risk factors
International PCOS Guidelines (2018)	All women with PCOS, particularly if overweight or obese	OGTT	At baseline and at least every 3 years
Endocrine Society	Women with PCOS and BMI ≥25 kg/m² or additional risk factors	OGTT	At baseline and periodically thereafter

Notably, the Endocrine Society and the 2018 International PCOS Guidelines emphasize the use of OGTT due to its superior sensitivity compared to HbA1c. PCOS, polycystic ovary syndrome; HbA1c, glycated hemoglobin; OGTT, oral glucose tolerance test.

As for adolescents, a growing body of literature emphasizes the need for systematic metabolic assessment at diagnosis. The international evidence-based guideline for the assessment and management of PCOS funded by the Australian National Health and Medical Research Council (NHMRC) in partnership with the European Society of Human Reproduction and Embryology (ESHRE) and the American Society for Reproductive Medicine (ASRM) suggest that baseline evaluation for adolescents of all body sizes should include blood pressure measurement, fasting lipid profile, and assessment of glucose metabolism through either fasting glucose, HbA1c, or preferably an OGTT when feasible (14). OGTT is considered the gold standard as multiple studies have shown that HbA1c alone underestimates the prevalence of impaired glucose tolerance in adolescents with PCOS (83). However, both have strengths and weaknesses, and because OGTT is time-consuming and often poorly tolerated in pediatric settings, a two-step approach could be an initial screening with HbA1c and/or fasting glucose, followed by OGTT in those with abnormal results or carrying additional risk factors such as obesity, family history of T2D, or clinical evidence of insulin resistance (acanthosis nigricans, rapid weight gain) (12). The recommended frequency of screening in adolescents also differs by risk profile. Adolescents with obesity, positive family history of diabetes, or metabolic abnormalities at baseline should undergo annual glucose testing, while those at lower risk could be re-evaluated every 2 to 3 years (14). Lipid screening should be performed at baseline and subsequently repeated every 2 to 3 years, whereas blood pressure should be assessed at each visit.

Additionally, aspartate aminotransferase (ALT) dosage is not routinely included in PCOS screening but could be included in PCOS screening. ALT is an enzyme involved in amino acid metabolism that is primarily localized in the liver, and its presence in the bloodstream reflects hepatocellular injury. Among the liver enzymes, ALT is considered the most specific marker of hepatocellular damage, and for this reason it has long been used in both clinical practice and research as a meter of liver health. From a practical perspective, ALT measurement is reasonably priced, widely available, and performed routinely in virtually all clinical laboratories. It requires only standard blood draw, and can be repeated at low cost, which makes it a feasible tool for longitudinal monitoring even in young populations. The relevance of ALT in adolescents with PCOS lies in its relationship with metabolic risk. Several studies have shown that ALT levels may be elevated in adolescents with PCOS, even in those who are not overtly obese, suggesting subclinical hepatic involvement and pointing to ALT as a potential early biomarker of metabolic complication (84). ALT is not a perfect marker, given its limited sensitivity and specificity and the need for lower pediatric cut-offs, but its use in adolescents with PCOS remains justified: testing is simple, inexpensive, and easily incorporated into routine panels, and results provide complementary information on hepatic involvement, a relevant index of higher metabolic risk.

## 7 Conclusions

PCOS represents a complex condition at the intersection of reproductive and metabolic health, with insulin resistance playing a pivotal role in its pathogenesis. In adolescents and young women, PCOS often coexists with features of the metabolic syndrome, including obesity, dyslipidemia, and impaired glucose tolerance, contributing to an increased lifetime risk of T2D and cardiovascular disease. While traditionally associated with obesity, it is now evident that insulin resistance and some features of metabolic syndrome can also be found in lean individuals with PCOS, suggesting that the metabolic abnormalities play a central role in the syndrome’s pathophysiology, independently of excess weight and adiposity. These findings reinforce the need for comprehensive evaluation of all patients with PCOS, starting from an early age and regardless of their body weight, and that can happen through metabolic screening and individualized risk assessment. Given the strong dimension of PCOS and the evidence that insulin resistance may precede and drive the reproductive dysfunction, early identification and comprehensive management are essential.

Current guidelines emphasize the importance of using OGTT over HbA1c or fasting glucose, given the poor sensitivity of HbA1c in identifying dysglycemia. Periodic OGTT is recommended even in the absence of classical risk factors, and should be complemented by a broader metabolic assessment, including lipid profile and liver enzymes to screen for conditions such as NAFLD. Despite increasing evidence of early onset cardiometabolic abnormalities, screening recommendations for adolescents with PCOS remain inconsistent. Incorporating both HbA1c and ALT into routine screening protocols may improve early risk identification, particularly in pediatric populations where OGTT may be less feasible.

Finally, in line with the results of recent major international engagement, we also support the proposal for a name change for PCOS. The current terminology emphasizes the ovarian aspects, contributing to a lack of awareness in both patients and some health professionals regarding the broad range of PCOS manifestations (metabolic, reproductive, psychological, cardiovascular, dermatological…) and contributing to delayed or missed diagnosis. A revised name could help improve early recognition and foster a more precise understanding of the complex and systemic nature of the disorder.

## References

[1] Bozdag G Mumusoglu S Zengin D Karabulut E Yildiz BO. The prevalence and phenotypic features of polycystic ovary syndrome: a systematic review and meta-analysis. Hum Reprod Oxf Engl. (2016) 31:2841–55. doi: 10.1093/humrep/dew218, PMID: 27664216

[2] Rotterdam ESHRE/ASRM-Sponsored PCOS consensus workshop group. Revised 2003 consensus on diagnostic criteria and long-term health risks related to polycystic ovary syndrome (PCOS). Hum Reprod Oxf Engl. (2004) 19:41–7. doi: 10.1016/j.fertnstert.2003.10.004, PMID: 14688154

[3] Carmina E Oberfield SE Lobo RA. The diagnosis of polycystic ovary syndrome in adolescents. Am J Obstet Gynecol. (2010) 203:201.e1–5. doi: 10.1016/j.ajog.2010.03.008, PMID: 20435290

[4] Legro RS Arslanian SA Ehrmann DA Hoeger KM Murad MH Pasquali R. Diagnosis and treatment of polycystic ovary syndrome: an endocrine society clinical practice guideline. J Clin Endocrinol Metab. (2013) 98:4565–92. doi: 10.1210/jc.2013-2350, PMID: 24151290 PMC5399492

[5] Bahri Khomami M Teede HJ Joham AE Moran LJ Piltonen TT Boyle JA. Clinical management of pregnancy in women with polycystic ovary syndrome: An expert opinion. Clin Endocrinol (Oxf). (2022) 97:227–36. doi: 10.1111/cen.14723, PMID: 35383999 PMC9544149

[6] Palomba S de Wilde MA Falbo A Koster MPH La Sala GB Fauser BCJM. Pregnancy complications in women with polycystic ovary syndrome. Hum Reprod Update. (2015) 21:575–92. doi: 10.1093/humupd/dmv029, PMID: 26117684

[7] Moran LJ Misso ML Wild RA Norman RJ. Impaired glucose tolerance, type 2 diabetes and metabolic syndrome in polycystic ovary syndrome: a systematic review and meta-analysis. Hum Reprod Update. (2010) 16:347–63. doi: 10.1093/humupd/dmq001, PMID: 20159883

[8] Ding T Hardiman PJ Petersen I Wang FF Qu F Baio G. The prevalence of polycystic ovary syndrome in reproductive-aged women of different ethnicity: a systematic review and meta-analysis. Oncotarget. (2017) 8:96351–8. doi: 10.18632/oncotarget.19180, PMID: 29221211 PMC5707105

[9] Naz MSG Tehrani FR Majd HA Ahmadi F Ozgoli G Fakari FR. The prevalence of polycystic ovary syndrome in adolescents: A systematic review and meta-analysis. Int J Reprod Biomed. (2019) 17:533–42. doi: 10.18502/ijrm.v17i8.4818, PMID: 31583370 PMC6745085

[10] Anagnostis P Paparodis RD Bosdou JK Bothou C Macut D Goulis DG. Risk of type 2 diabetes mellitus in polycystic ovary syndrome is associated with obesity: a meta-analysis of observational studies. Endocrine. (2021) 74:245–53. doi: 10.1007/s12020-021-02801-2, PMID: 34176074

[11] Toosy S Sodi R Pappachan JM. Lean polycystic ovary syndrome (PCOS): an evidence-based practical approach. J Diabetes Metab Disord. (2018) 17:277–85. doi: 10.1007/s40200-018-0371-5, PMID: 30918863 PMC6405408

[12] Ibáñez L de Zegher F. Polycystic ovary syndrome in adolescent girls. Pediatr Obes. (2020) 15:e12586. doi: 10.1111/ijpo.12586, PMID: 31663293

[13] Moran LJ Deeks AA Gibson-Helm ME Teede HJ. Psychological parameters in the reproductive phenotypes of polycystic ovary syndrome. Hum Reprod Oxf Engl. (2012) 27:2082–8. doi: 10.1093/humrep/des114, PMID: 22493025

[14] Teede HJ Tay CT Laven JJE Dokras A Moran LJ Piltonen TT. Recommendations from the 2023 international evidence-based guideline for the assessment and management of polycystic ovary syndrome. J Clin Endocrinol Metab. (2023) 108:2447–69. doi: 10.1210/clinem/dgad463, PMID: 37580314 PMC10505534

[15] Behera M Price T Walmer D. Estrogenic ovulatory dysfunction or functional female hyperandrogenism: an argument to discard the term polycystic ovary syndrome. Fertil Steril. (2006) 86:1292–5. doi: 10.1016/j.fertnstert.2006.06.048, PMID: 17070182

[16] Dunaif A Fauser BCJM. Renaming PCOS–a two-state solution. J Clin Endocrinol Metab. (2013) 98:4325–8. doi: 10.1210/jc.2013-2040, PMID: 24009134 PMC3816269

[17] Teede HJ Moran LJ Morman R Gibson M Dokras A Berry L. Polycystic ovary syndrome perspectives from patients and health professionals on clinical features, current name, and renaming: a longitudinal international online survey. EClinicalMedicine. (2025) 84:103287. doi: 10.1016/j.eclinm.2025.103287, PMID: 40687737 PMC12273733

[18] New name_PCOS (2025). Available online at: https://monash.az1.qualtrics.com/jfe/form/SV_eWKSMw04xjUDNmS (Accessed August 31, 2025).

[19] Genazzani AD Genazzani AR. Polycystic ovary syndrome as metabolic disease: new insights on insulin resistance. TouchREVIEWS Endocrinol. (2023) 19:71–7. doi: 10.17925/EE.2023.19.1.71, PMID: 37313240 PMC10258623

[20] Brennan K Huang A Azziz R. Dehydroepiandrosterone sulfate and insulin resistance in patients with polycystic ovary syndrome. Fertil Steril. (2009) 91:1848–52. doi: 10.1016/j.fertnstert.2008.02.101, PMID: 18439591 PMC2691796

[21] Kelsey MM Zeitler PS. Insulin resistance of puberty. Curr Diabetes Rep. (2016) 16:64. doi: 10.1007/s11892-016-0751-5, PMID: 27179965

[22] Huang X Liu G Guo J Su Z. The PI3K/AKT pathway in obesity and type 2 diabetes. Int J Biol Sci. (2018) 14:1483–96. doi: 10.7150/ijbs.27173, PMID: 30263000 PMC6158718

[23] Copps KD White MF. Regulation of insulin sensitivity by serine/threonine phosphorylation of insulin receptor substrate proteins IRS1 and IRS2. Diabetologia. (2012) 55:2565–82. doi: 10.1007/s00125-012-2644-8, PMID: 22869320 PMC4011499

[24] Kopprasch S Srirangan D Bergmann S Graessler J Schwarz PEH Bornstein SR. Association between systemic oxidative stress and insulin resistance/sensitivity indices - the PREDIAS study. Clin Endocrinol (Oxf). (2016) 84:48–54. doi: 10.1111/cen.12811, PMID: 25940301

[25] Diamanti-Kandarakis E Dunaif A. Insulin resistance and the polycystic ovary syndrome revisited: an update on mechanisms and implications. Endocr Rev. (2012) 33:981–1030. doi: 10.1210/er.2011-1034, PMID: 23065822 PMC5393155

[26] Lewy VD Danadian K Witchel SF Arslanian S. Early metabolic abnormalities in adolescent girls with polycystic ovarian syndrome. J Pediatr. (2001) 138:38–44. doi: 10.1067/mpd.2001.109603, PMID: 11148510

[27] American Diabetes Association. 13. Children and adolescents: standards of medical care in diabetes-2019. Diabetes Care. (2019) 42:S148–64. doi: 10.2337/dc19-S013, PMID: 30559239

[28] TODAY Study Group Zeitler P Hirst K Pyle L Linder B Copeland K. A clinical trial to maintain glycemic control in youth with type 2 diabetes. N Engl J Med. (2012) 366:2247–56. doi: 10.1056/NEJMoa1109333, PMID: 22540912 PMC3478667

[29] Alberti KG Zimmet PZ. Definition, diagnosis and classification of diabetes mellitus and its complications. Part 1: diagnosis and classification of diabetes mellitus provisional report of a WHO consultation. Diabetes Med J Br Diabetes Assoc. (1998) 15:539–53. doi: 10.1002/(SICI)1096-9136(199807)15:7<539::AID-DIA668>3.0.CO;2-S, PMID: 9686693

[30] National Cholesterol Education Program (NCEP) Expert Panel on Detection, Evaluation, and Treatment of High Blood Cholesterol in Adults (Adult Treatment Panel III). Third Report of the National Cholesterol Education Program (NCEP) Expert Panel on Detection, Evaluation, and Treatment of High Blood Cholesterol in Adults (Adult Treatment Panel III) final report. Circulation. (2002) 106:3143–421. doi: 10.1161/circ.106.25.3143, PMID: 12485966

[31] Alberti KGMM Zimmet P Shaw J. Metabolic syndrome–a new world-wide definition. A Consensus Statement from the International Diabetes Federation. Diabetes Med J Br Diabetes Assoc. (2006) 23:469–80. doi: 10.1111/j.1464-5491.2006.01858.x, PMID: 16681555

[32] Rahmanpour H Jamal L Mousavinasab SN Esmailzadeh A Azarkhish K. Association between polycystic ovarian syndrome, overweight, and metabolic syndrome in adolescents. J Pediatr Adolesc Gynecol. (2012) 25:208–12. doi: 10.1016/j.jpag.2012.02.004, PMID: 22578482

[33] Carmina E Napoli N Longo RA Rini GB Lobo RA. Metabolic syndrome in polycystic ovary syndrome (PCOS): lower prevalence in southern Italy than in the USA and the influence of criteria for the diagnosis of PCOS. Eur J Endocrinol. (2006) 154:141–5. doi: 10.1530/eje.1.02058, PMID: 16382003

[34] Kakoly NS Earnest A Teede HJ Moran LJ Joham AE. The impact of obesity on the incidence of type 2 diabetes among women with polycystic ovary syndrome. Diabetes Care. (2019) 42:560–7. doi: 10.2337/dc18-1738, PMID: 30705063

[35] Zhu S Zhang B Jiang X Li Z Zhao S Cui L. Metabolic disturbances in non-obese women with polycystic ovary syndrome: a systematic review and meta-analysis. Fertil Steril. (2019) 111:168–77. doi: 10.1016/j.fertnstert.2018.09.013, PMID: 30611404

[36] Ryu KJ Kim MS Kim HK Kim YJ Yi KW Shin JH. Risk of type 2 diabetes is increased in nonobese women with polycystic ovary syndrome: the National Health Insurance Service-National Sample Cohort Study. Fertil Steril. (2021) 115:1569–75. doi: 10.1016/j.fertnstert.2020.12.018, PMID: 33509630

[37] Qin J Li R Raes J Arumugam M Burgdorf KS Manichanh C. A human gut microbial gene catalogue established by metagenomic sequencing. Nature. (2010) 464:59–65. doi: 10.1038/nature08821, PMID: 20203603 PMC3779803

[38] Gomez-Arango LF Barrett HL McIntyre HD Callaway LK Morrison M Dekker Nitert M. Connections between the gut microbiome and metabolic hormones in early pregnancy in overweight and obese women. Diabetes. (2016) 65:2214–23. doi: 10.2337/db16-0278, PMID: 27217482

[39] Giampaolino P Foreste V Di Filippo C Gallo A Mercorio A Serafino P. Microbiome and PCOS: state-of-art and future aspects. Int J Mol Sci. (2021) 22:2048. doi: 10.3390/ijms22042048, PMID: 33669557 PMC7922491

[40] Liu R Zhang C Shi Y Zhang F Li L Wang X. Dysbiosis of gut microbiota associated with clinical parameters in polycystic ovary syndrome. Front Microbiol. (2017) 8:324. doi: 10.3389/fmicb.2017.00324, PMID: 28293234 PMC5328957

[41] Qi X Yun C Pang Y Qiao J. The impact of the gut microbiota on the reproductive and metabolic endocrine system. Gut Microbes. (2021) 13:1–21. doi: 10.1080/19490976.2021.1894070, PMID: 33722164 PMC7971312

[42] Zhou M Yu J Li X Ruan Z Yu S. Role of the gut microbiota and innate immunity in polycystic ovary syndrome: Current updates and future prospects. J Cell Mol Med. (2024) 28:e18258. doi: 10.1111/jcmm.18258, PMID: 38546608 PMC10977384

[43] Kukaev E Kirillova E Tokareva A Rimskaya E Starodubtseva N Chernukha G. Impact of gut microbiota and SCFAs in the pathogenesis of PCOS and the effect of metformin therapy. Int J Mol Sci. (2024) 25:10636. doi: 10.3390/ijms251910636, PMID: 39408965 PMC11477200

[44] Chen T Long W Zhang C Liu S Zhao L Hamaker BR. Fiber-utilizing capacity varies in Prevotella- versus Bacteroides-dominated gut microbiota. Sci Rep. (2017) 7:2594. doi: 10.1038/s41598-017-02995-4, PMID: 28572676 PMC5453967

[45] Tabrizi R Ostadmohammadi V Akbari M Lankarani KB Vakili S Peymani P. The effects of probiotic supplementation on clinical symptom, weight loss, glycemic control, lipid and hormonal profiles, biomarkers of inflammation, and oxidative stress in women with polycystic ovary syndrome: a systematic review and meta-analysis of randomized controlled trials. Probiotics Antimicrob Proteins. (2022) 14:1–14. doi: 10.1007/s12602-019-09559-0, PMID: 31165401

[46] Li Y Tan Y Xia G Shuai J. Effects of probiotics, prebiotics, and synbiotics on polycystic ovary syndrome: a systematic review and meta-analysis. Crit Rev Food Sci Nutr. (2023) 63:522–38. doi: 10.1080/10408398.2021.1951155, PMID: 34287081

[47] Ahmadi S Jamilian M Karamali M Tajabadi-Ebrahimi M Jafari P Taghizadeh M. Probiotic supplementation and the effects on weight loss, glycaemia and lipid profiles in women with polycystic ovary syndrome: a randomized, double-blind, placebo-controlled trial. Hum Fertil Camb Engl. (2017) 20:254–61. doi: 10.1080/14647273.2017.1283446, PMID: 28142296

[48] Statement of Retraction: Probiotic supplementation and the effects on weight loss, glycaemia and lipid profiles in women with polycystic ovary syndrome: a randomised, double-blind, placebo-controlled trial. Hum Fertil Camb Engl. (2024) 27:2373595. doi: 10.1080/14647273.2024.2373595, PMID: 38953512

[49] Qi X Yun C Sun L Xia J Wu Q Wang Y. Gut microbiota-bile acid-interleukin-22 axis orchestrates polycystic ovary syndrome. Nat Med. (2019) 25:1225–33. doi: 10.1038/s41591-019-0509-0, PMID: 31332392 PMC7376369

[50] DeFronzo RA Tobin JD Andres R. Glucose clamp technique: a method for quantifying insulin secretion and resistance. Am J Physiol. (1979) 237:E214–223. doi: 10.1152/ajpendo.1979.237.3.E214, PMID: 382871

[51] Amisi CA. Markers of insulin resistance in Polycystic ovary syndrome women: An update. World J Diabetes. (2022) 13:129–49. doi: 10.4239/wjd.v13.i3.129, PMID: 35432749 PMC8984569

[52] Amini A Soltanian N Iraj B Askari G Ebneyamin S Ghias M. Association of wrist circumference with cardio metabolic risk factors. JPMA J Pak Med Assoc. (2012) 62:S34–36., PMID: 22768455

[53] Stolk RP Van Daele PL Pols HA Burger H Hofman A Birkenhäger JC. Hyperinsulinemia and bone mineral density in an elderly population: The Rotterdam Study. Bone. (1996) 18:545–9. doi: 10.1016/8756-3282(96)00079-8, PMID: 8805995

[54] Lerchbaum E Schwetz V Giuliani A Obermayer-Pietsch B. Assessment of glucose metabolism in polycystic ovary syndrome: HbA1c or fasting glucose compared with the oral glucose tolerance test as a screening method. Hum Reprod Oxf Engl. (2013) 28:2537–44. doi: 10.1093/humrep/det255, PMID: 23756702

[55] Ortiz-Flores AE Luque-Ramírez M Fernández-Durán E Alvarez-Blasco F Escobar-Morreale HF. Diagnosis of disorders of glucose tolerance in women with polycystic ovary syndrome (PCOS) at a tertiary care center: fasting plasma glucose or oral glucose tolerance test? Metabolism. (2019) 93:86–92. doi: 10.1016/j.metabol.2019.01.015, PMID: 30710572

[56] Gupta J Antal Z Mauer E Gerber LM An A Censani M. Dysglycemia screening with oral glucose tolerance test in adolescents with polycystic ovary syndrome and relationship with obesity. BMC Endocr Disord. (2022) 22:180. doi: 10.1186/s12902-022-01098-0, PMID: 35842601 PMC9288674

[57] Coles N Bremer K Kives S Zhao X Hamilton J. Utility of the oral glucose tolerance test to assess glucose abnormalities in adolescents with polycystic ovary syndrome. J Pediatr Adolesc Gynecol. (2016) 29:48–52. doi: 10.1016/j.jpag.2015.06.004, PMID: 26358939

[58] Matthews DR Hosker JP Rudenski AS Naylor BA Treacher DF Turner RC. Homeostasis model assessment: insulin resistance and beta-cell function from fasting plasma glucose and insulin concentrations in man. Diabetologia. (1985) 28:412–9. doi: 10.1007/BF00280883, PMID: 3899825

[59] Tosi F Bonora E Moghetti P. Insulin resistance in a large cohort of women with polycystic ovary syndrome: a comparison between euglycaemic-hyperinsulinaemic clamp and surrogate indexes. Hum Reprod Oxf Engl. (2017) 32:2515–21. doi: 10.1093/humrep/dex308, PMID: 29040529

[60] DeUgarte CM Bartolucci AA Azziz R. Prevalence of insulin resistance in the polycystic ovary syndrome using the homeostasis model assessment. Fertil Steril. (2005) 83:1454–60. doi: 10.1016/j.fertnstert.2004.11.070, PMID: 15866584

[61] Wei HJ Young R Kuo IL Liaw CM Chiang HS Yeh CY. Prevalence of insulin resistance and determination of risk factors for glucose intolerance in polycystic ovary syndrome: a cross-sectional study of Chinese infertility patients. Fertil Steril. (2009) 91:1864–8. doi: 10.1016/j.fertnstert.2008.02.168, PMID: 18565519

[62] Morciano A Romani F Sagnella F Scarinci E Palla C Moro F. Assessment of insulin resistance in lean women with polycystic ovary syndrome. Fertil Steril. (2014) 102:250–6. doi: 10.1016/j.fertnstert.2014.04.004, PMID: 24825420

[63] Song DK Hong YS Sung YA Lee H. Insulin resistance according to β-cell function in women with polycystic ovary syndrome and normal glucose tolerance. PloS One. (2017) 12:e0178120. doi: 10.1371/journal.pone.0178120, PMID: 28542421 PMC5444780

[64] Duncan MH Singh BM Wise PH Carter G Alaghband-Zadeh J. A simple measure of insulin resistance. Lancet Lond Engl. (1995) 346:120–1. doi: 10.1016/S0140-6736(95)92143-5, PMID: 7603193

[65] Perseghin G Caumo A Caloni M Testolin G Luzi L. Incorporation of the fasting plasma FFA concentration into QUICKI improves its association with insulin sensitivity in nonobese individuals. J Clin Endocrinol Metab. (2001) 86:4776–81. doi: 10.1210/jcem.86.10.7902, PMID: 11600540

[66] Matsuda M DeFronzo RA. Insulin sensitivity indices obtained from oral glucose tolerance testing: comparison with the euglycemic insulin clamp. Diabetes Care. (1999) 22:1462–70. doi: 10.2337/diacare.22.9.1462, PMID: 10480510

[67] Rizzo M Tyndall EK Frontoni S Jacoangeli F Sarlo F Panebianco F. Rapid and easy assessment of insulin resistance contributes to early detection of polycystic ovary syndrome. J Endocrinol Invest. (2013) 36:527–30. doi: 10.3275/8947, PMID: 23612476

[68] Nowak-Ciołek M Stachowiak JJ Krok K Sokal J Malczyk Ż Skrzyńska K. Adolescent PCOS and long-term metabolic risk: insights from triglycerides to high-density lipoprotein cholesterol ratio and high-density lipoprotein cholesterol profiles. Front Endocrinol. (2025) 16:1579217. doi: 10.3389/fendo.2025.1579217, PMID: 40529833 PMC12170654

[69] Wang L Cong HL Zhang JX Hu YC Wei A Zhang YY. Triglyceride-glucose index predicts adverse cardiovascular events in patients with diabetes and acute coronary syndrome. Cardiovasc Diabetol. (2020) 19:80. doi: 10.1186/s12933-020-01054-z, PMID: 32534586 PMC7293784

[70] Javidan A Azarboo A Jalali S Fallahtafti P Moayyed S Ghaemi M. The association between triglyceride-glucose index and polycystic ovary syndrome: a systematic review and meta-analysis across different populations. J Ovarian Res. (2025) 18:163. doi: 10.1186/s13048-025-01717-z, PMID: 40713814 PMC12291403

[71] Taşkömür AT Erten Ö. Relationship of inflammatory and metabolic parameters in adolescents with PCOS: BMI matched case-control study. Arch Endocrinol Metab. (2022) 66:372–81. doi: 10.20945/2359-3997000000497, PMID: 35657129 PMC9832847

[72] Gozashti MH Gholamhosseinian A Musavi F Mashrouteh M. Relationship between serum cystatin C and polycystic ovary syndrome. Iran J Reprod Med. (2013) 11:71–6., PMID: 24639696 PMC3941379

[73] Burrows R Correa-Burrows P Reyes M Blanco E Albala C Gahagan S. Healthy Chilean adolescents with HOMA-IR ≥ 2.6 have increased cardiometabolic risk: association with genetic, biological, and environmental factors. J Diabetes Res. (2015) 2015:783296. doi: 10.1155/2015/783296, PMID: 26273675 PMC4530255

[74] Sun Y Ji H Sun W An X Lian F. Triglyceride glucose (TyG) index: A promising biomarker for diagnosis and treatment of different diseases. Eur J Intern Med. (2025) 131:3–14. doi: 10.1016/j.ejim.2024.08.026, PMID: 39510865

[75] Kheirollahi A Teimouri M Karimi M Vatannejad A Moradi N Borumandnia N. Evaluation of lipid ratios and triglyceride-glucose index as risk markers of insulin resistance in Iranian polycystic ovary syndrome women. Lipids Health Dis. (2020) 19:235. doi: 10.1186/s12944-020-01410-8, PMID: 33161896 PMC7648985

[76] Rodriguez J Cordaillat-Simmons M Badalato N Berger B Breton H de Lahondès R. Microbiome testing in Europe: navigating analytical, ethical and regulatory challenges. Microbiome. (2024) 12:258. doi: 10.1186/s40168-024-01991-x, PMID: 39695869 PMC11657758

[77] Agrawal A Dave A Jaiswal A. Type 2 diabetes mellitus in patients with polycystic ovary syndrome. Cureus. (2023) 15:e46859. doi: 10.7759/cureus.46859, PMID: 37954695 PMC10637759

[78] Liu Z Zhang Y Graham S Wang X Cai D Huang M. Causal relationships between NAFLD, T2D and obesity have implications for disease subphenotyping. J Hepatol. (2020) 73:263–76. doi: 10.1016/j.jhep.2020.03.006, PMID: 32165250 PMC7371536

[79] Genua I Iruzubieta P Rodríguez-Duque JC Pérez A Crespo J. NAFLD and type 2 diabetes: A practical guide for the joint management. Gastroenterol Hepatol. (2023) 46:815–25. doi: 10.1016/j.gastrohep.2022.12.002, PMID: 36584750

[80] Schroeder BM American College of Obstetricians and Gynecologists. ACOG releases guidelines on diagnosis and management of polycystic ovary syndrome. Am Fam Physician. (2003) 67:1619–20:1622., PMID: 12722867

[81] Preventive Services Task Force US Davidson KW Barry MJ Mangione CM Cabana M Caughey AB. Screening for prediabetes and type 2 diabetes: US preventive services task force recommendation statement. JAMA. (2021) 326:736–43. doi: 10.1001/jama.2021.12531, PMID: 34427594

[82] Dumesic DA Oberfield SE Stener-Victorin E Marshall JC Laven JS Legro RS. Scientific statement on the diagnostic criteria, epidemiology, pathophysiology, and molecular genetics of polycystic ovary syndrome. Endocr Rev. (2015) 36:487–525. doi: 10.1210/er.2015-1018, PMID: 26426951 PMC4591526

[83] Gooding HC Milliren C St Paul M Mansfield MJ DiVasta A. Diagnosing dysglycemia in adolescents with polycystic ovary syndrome. J Adolesc Health Off Publ Soc Adolesc Med. (2014) 55:79–84. doi: 10.1016/j.jadohealth.2013.12.020, PMID: 24560306

[84] Liu C Liu K Zhao X Zhu J Liu Y Hao L. The associations between alanine aminotransferase and other biochemical parameters in lean PCOS. Reprod Sci. (2022) 30:633–41. doi: 10.1007/s43032-022-01030-w, PMID: 35864417 PMC9988735

